# Characteristics of the Frustrated Lewis Pairs (FLPs) on the Surface of Albite and the Corresponding Mechanism of H_2_ Activation

**DOI:** 10.1002/open.202300058

**Published:** 2023-10-06

**Authors:** Yannan Zhou, Xuegang Luo

**Affiliations:** ^1^ Research Center of Laser Fusion China Academy of Engineering Physics Mianyang Sichuan 621900 P. R. China; ^2^ Institute of Salt Lakes Chinese Academy of Science Xining Qinghai 810008 P. R. China; ^3^ Engineering Research Center of Biomass Materials Ministry of Education Mianyang Sichuan 621010 P. R. China

**Keywords:** Albite, Frustrated Lewis pairs, Hydrogen, Density functional theory, Frontier orbital theory

## Abstract

The characteristics of frustrated Lewis pairs (FLPs) on albite surfaces were analyzed with density functional theory, and the reaction mechanism for H_2_ activation by the FLPs was studied. The results show that albite is an ideal substrate material with FLPs, and its (001) and (010) surfaces have the typical characteristics of FLPs. In the case of H_2_ activation, the interaction between the HOMO of H_2_ and the SOMO of the Lewis base and the electron acceptance characteristics of the Lewis acid are the key factors. In fact, the activation energy of H_2_ is the required activation energy from the ground state to the excited state, and once the excited state is produced, the dissociative adsorption of H_2_ will occur directly. This study provides a new ideas and a reference for research on the construction of novel solid FLPs catalysts using ultramicro channel materials.

## Graphical abstract

In the reaction of H_2_ activation, the interaction between the HOMO of H_2_ and the SOMO of LB and the electron acceptance characteristics of LA are the key factors. The activation energy of H_2_ is the required activation energy from the ground state to the excited state, once the excited state is produced, dissociation adsorption of H_2_ will occur directly.

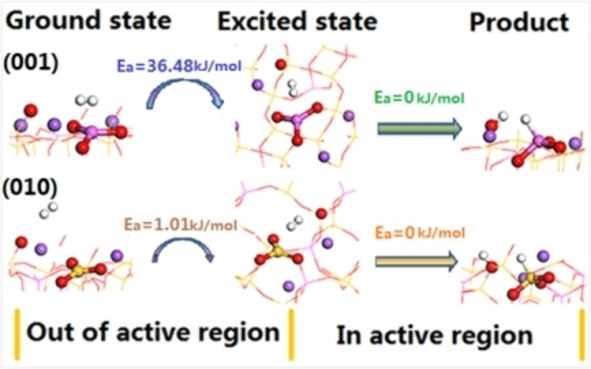

## Introduction

Frustrated Lewis pairs (FLPs) are nonmetallic organic catalysts used in homogeneous catalysis, and they exhibit high activities in activating inert small molecules such as H_2_ and have attracted increasing attention. However, homogeneous FLPs have some disadvantages, such as poor stability and difficult recycling, which restrict their use in large‐scale catalytic reactions. Therefore, the development of all‐solid state FLP catalysts has become more attractive. In constructing all‐solid state FLPs, the first requirement is that the solid surface should contain both Lewis acids and Lewis bases. Second, they must be located at the right distance from each other. FLPs can interact with each other but cannot form bonded adducts. To date, the materials used to construct all‐solid state FLPs include hydroxylated indium oxide,[^1^, ^2^, ^3^, ^4^, ^5^] doped graphene,[^6^, ^7^] metal‐organic frameworks,[^8^, ^9^, ^10^] boron/aluminum‐doped two‐dimensional phosphorene,^[11]^ among others. Although FLPs constructed from these materials are active in hydrogenation, they are plagued by problems such as difficult preparation methods or restricted catalytic activities. The challenge is that there are few effective methods for accurate construction of FLP sites, and very few successful cases have been reported. Y. Q. Qu and C. R. Chang et al.^[12]^ constructed FLPs by regulating CeO_2_ oxygen vacancies and realized the activation of H_2_, CO_2_ and other small molecules. J. L. Zhang et al.^[13]^ constructed an anatase‐TiO_2_‐based structure by doping with Ga^3+^ to achieve photocatalytic activation of C−H bonds with FLPs. The goal is to find systems and strategies with appropriate steric encumbrance. A. M. Zheng et al.^[14]^ constructed FLPs in zeolite molecular sieve cages and believed that formation of the sterically encumbered FLPs depended on obstructions from pore size limitation. S. Q. Ma et al.^[15]^ proposed that the use of porous structural materials to construct FLPs is a very promising direction but also provided new challenges. As a natural mineral, albite has a remarkable ultramicro channel structure, which should have research value, and it also provides a new strategy for the construction of new solid FLPs. In this article, based on Lewis acid‐base theory and the definition of FLPs, the Lewis acid base sites on the albite surface were analyzed theoretically, the acid‐base types were characterized with IR probe molecules, and the effect and reaction mechanism of H_2_ activation were studied and analyzed to provide reference information for the construction of solid FLPs.

## Results and Discussion

### Theoretical analyses of FLP sites on albite surfaces

Albite is a common feldspar mineral with the chemical formula NaAlSi_3_O_8_, and its structure comprises a three‐dimensional framework of corner‐sharing SiO_4_ and AlO_4_ tetrahedra. Albite has two cleavage surfaces, (001) and (010), which are also the two commonly exposed surfaces.[^16^, ^17^] Generally, the surfaces of natural minerals are formed by bond breaking, which generates an imbalanced solid surface charge and leads to surface reactivity.^[18]^

**Figure 1:**
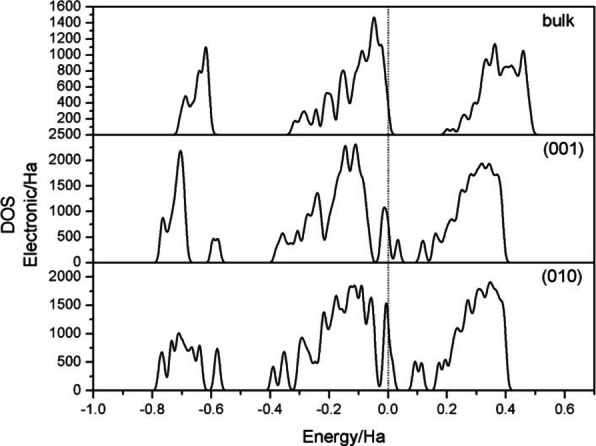
Total densities of states for bulk albite and the surfaces of albite.

The density of states at the Fermi level is important information^[12]^ reflecting the surface activity, and the higher the peak value is for the density of states is, the greater the reactivity. A density of states on the right side of the Fermi level represents the ability to gain electrons, and a density of states on the left side represents the ability to donate electrons. The closer the density of states is to the Fermi level, the greater the ability to gain or lose electrons and the more difficult it is to move away from the Fermi level.[^19^, ^20^, ^21^] To analyze the properties of the two surfaces, we calculated the electronic density of states for the bulk phase and the two surfaces (Figure 1).

The DOS for the bulk phase tended to zero at the Fermi level but did not reach zero, indicating that the system had nonlocal properties. Both the (001) and (010) surfaces showed local peaks at the Fermi level, indicating that the surfaces were reactive. Moreover, the peak value of the (010) surface was higher than that of the (001) surface, which also indicated that the (010) surface was more reactive than the (001) surface.

**Figure 2:**
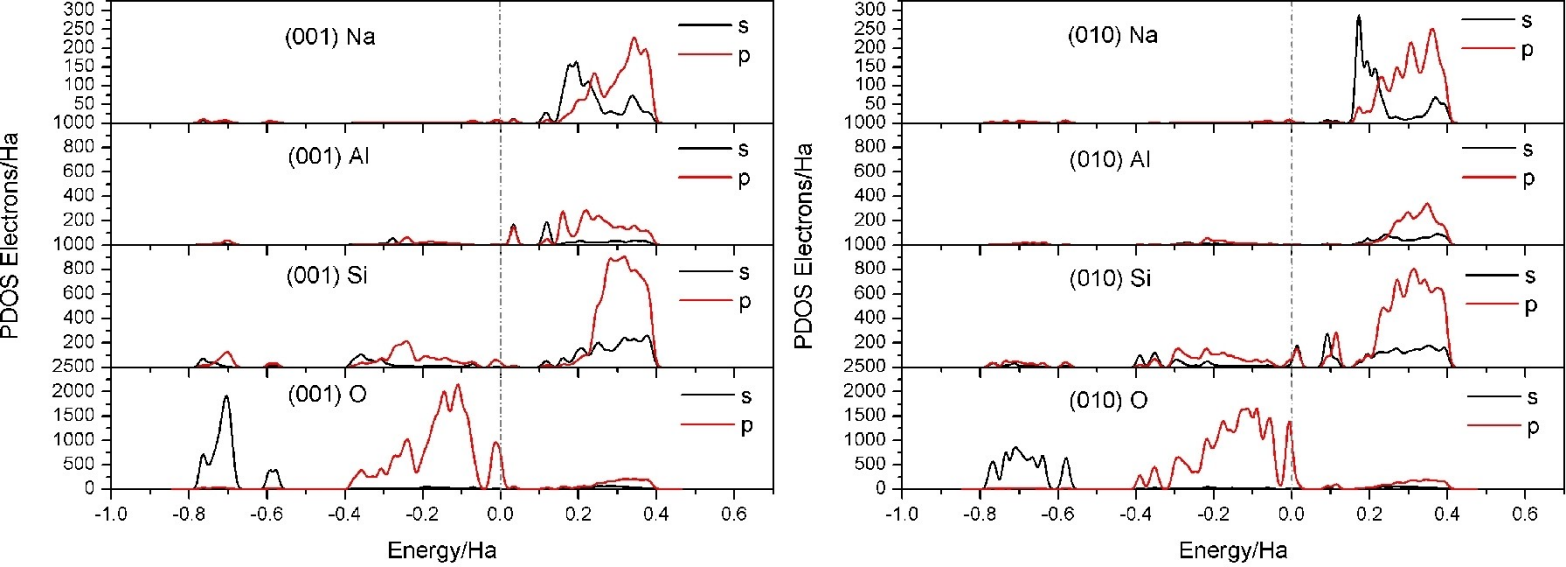
Partial density of states for Al, O, Si, and Na on the albite surface layer of FLPs.

Figure 2 shows the partial densities of Na, Al, Si and O on the two surfaces. The (001) density of states at the Fermi level was contributed by the 3 s and 3p orbitals of Al and the 2p orbitals of O, Al and O are reactive. Also, the (010) surface DOS resulting from the 3 s and 3p orbitals of Si and the 2p orbitals of O, Si and O are reactive. The position of the DOS resulting from Al and Si showed that Al was less likely to attract electrons than Si. These analyses showed that Al and O atoms exposed at the albite (001) surface layer were active sites, while the Si and O exposed at the albite (010) surface layer provided the active sites. This was mainly due to cleavage along the crystal surfaces. On the (001) surface, the Al−O−Si bonds perpendicular to the cleavage surface broke to form an Al−O bonds. At the (010) surface, the Si−O−Si bonds perpendicular to the cleavage surface broke to form Si−O bonds, and they were exposed on the surface layer and were coordinatively unsaturated.

**Figure 3:**
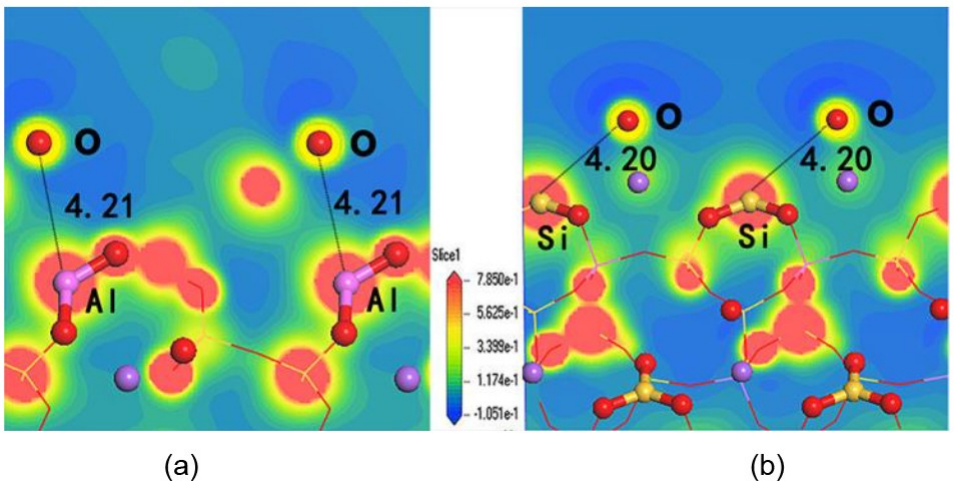
Charge density distributions on the surface of albite: (a) represents the (001) surface, and (b) represents the (010) surface.(the distance of O−Al and O−Si is 4.21 Å and 4.20 Å).

Figure 3 shows the charge densities of coordinatively unsaturated O, Si and Al on the (001) and (010) surfaces, and red corresponds to low electron density and blue corresponds to high electron density. Both Al and Si were located in a region of low electron density, indicating that they were electron‐deficient and Lewis‐acidic (LA). The O atoms exposed on the surface were in a region of high electron density, so they were electron‐rich and Lewis basic (LB). Whether on the (001) or (010) surface, neither the Lewis acids or bases were bonded, indicating that they were independent of each other and located on the surface of the skeleton, so they did not form acid‐base adducts. Because albite has an ultramicro channel structure, the apertures are generally smaller than 3 Å, which enables the formation of sterically encumbered crystals measuring 4.21 Å and 4.20 Å.[^12^, ^13^] The Lewis acid‐base pairs on the albite (001) and (010) surfaces satisfy the basic conditions for FLPs.

### Molecular characterization of surface acids and bases with IR probe

Important information can be obtained directly from theoretical calculations on surface acids and bases bonded to IR probe molecules. Due to the undulating and uneven surfaces of the ultramicro channel structure, the spaces around the surface acid sites are narrow, and the accessibility for pyridine is limited. Therefore, NH_3_ was selected as a probe molecule for acidic centers to identify the type of acid centers found on the albite surface.

**Figure 4:**
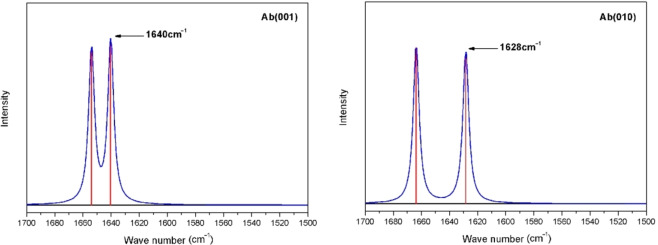
Infrared spectra of NH_3_ on the (001) and (010) surfaces.

The model for NH_3_ adsorption on two surface‐acidic centers was established, and the adsorption energies and IR spectra for NH_3_ adsorbed on the albite (001) and (010) surfaces were calculated. Figure 4 shows the IR spectra (IR) for NH_3_ probe molecules on the (001) and (010) surfaces. The IR peak near 1600–1640 cm^−1^ is the characteristic peak for NH_3_ adsorbed at the site of a Lewis acid.[^22^, ^23^, ^24^, ^25^] As shown in Figure 4, there were also peaks at 1640 cm^−1^ and 1628 cm^−1^ in the IR spectra of the (001) surface and (010) surface, indicating that the Al on the (001) surface and Si on the (010) surface were Lewis acid centers.

**Figure 5:**
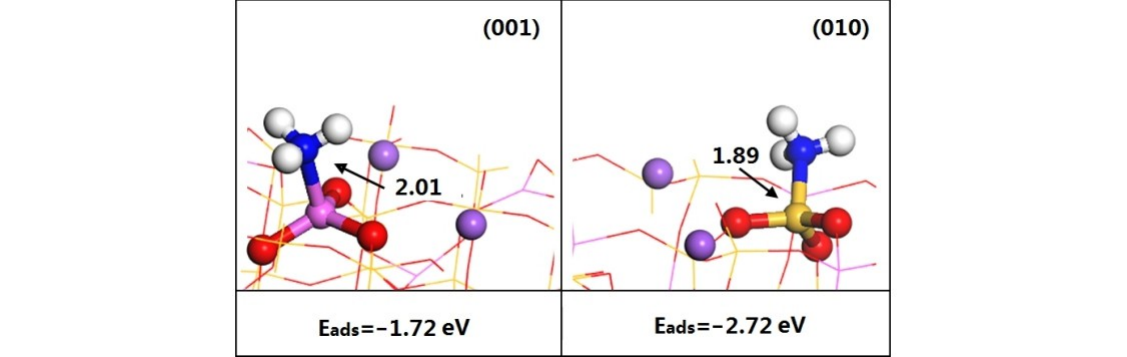
Stable adsorption configurations, adsorption energies and bond lengths for NH_3_ adsorbed on the (001) and (010) surfaces.(the bond length of N−Al and N−Si is 2.01 Å and 1.89 Å).

Figure 5 shows the configurations of NH_3_ adsorbed on the (001) and (010) surfaces. When the NH_3_ probe molecule was stably adsorbed at an Al site on the (001) surface or a Si site on the (010) surface, the N was bonded to the Al or Si, respectively, and the N−Al bond length was longer than that of the N−Si bond. Moreover, the energy calculated for NH_3_ adsorption on the (001) surface was −1.72 eV, which was lower than that of the (010) surface (−2.72 eV). Generally, there is a positive linear correlation between the capacity for NH_3_ adsorption at a Lewis acid site and the acidity of the Lewis acid site.^[26]^ The greater the adsorption energy for NH_3_, the higher the desorption temperature needed, and the stronger the acidity of the Lewis acid site. The Lewis acidity of the (010) surface was stronger than that of the (001) surface.

**Figure 6:**
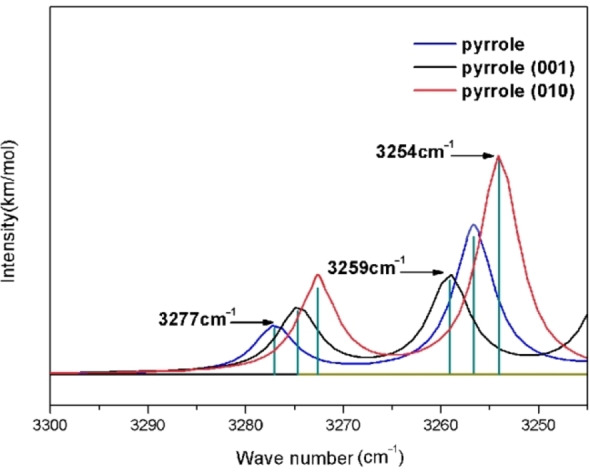
Infrared spectra of free pyrrole and pyrrole adsorbed on the two surfaces.

To characterize the Lewis base sites on the two surfaces, the configurations of pyrrole adsorbed on the two surface bases were established by using the infrared spectral data for pyrrole probe molecules adsorbed on the alkaline sites of molecular sieves. The energies for pyrrole adsorption on the albite (001) and (010) surfaces and the infrared spectra of the adsorption systems were calculated. Figure 6 shows the infrared peaks for pyrrole itself and for pyrrole adsorbed on the (001) and (010) surfaces. Generally, the IR peaks for pyrrole appear at 3182–3284 cm^−1^, and this is the characteristic peak indicating adsorption at a Lewis base site.^[27]^ As shown in Figure 6, there was also a characteristic peak at 3259 cm^−1^ for the (001) surface and at 3254 cm^−1^ for the (010) surface, indicating that the oxygens on the (001) and (010) surfaces are Lewis base centers. For the strength of the Lewis base, it could be confirmed with the infrared absorption frequency shift of the pyrrole N−H bond.^[25]^ The infrared peaks for pyrrole adsorbed on the (001) and (010) surfaces were shifted toward lower frequencies, and the frequency shift for the (010) surface was greater than that for the (001) surface, indicating that the Lewis basicity of the (010) surface was stronger than that of the (001) surface.

**Figure 7:**
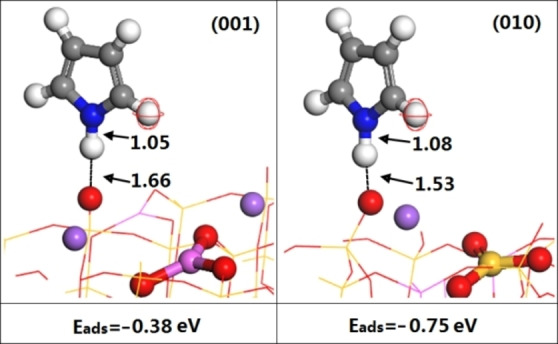
Stable adsorption configurations, adsorption energies and bond lengths for pyrrole on the (001) and (010) surfaces.(the distance of O−H on (001) and (010) is 1.66 Å and 1.53 Å, while the bond length of N−H on (001) and (010) is 1.05 Å and1.08 Å).

Figure 7 shows the configurations for pyrrole adsorbed on the (001) and (010) surfaces. The H−O distances of the (001) and (010) surfaces were 1.66 Å and 1.53 Å, respectively, and the pyrrole adsorption energies were −0.38 eV and −0.75 eV, respectively. A proton of the pyrrole molecule formed a hydrogen bond with the O atom of the Lewis base. We also found that the strengths of the Lewis base sites could be determined from the energy for pyrrole adsorption and the change in the N−H bond length. In other words, the larger the adsorption energy was, the longer the N−H bond and the stronger the basicity.

This was exactly the same as the conclusion reached from the magnitude of the frequency shift. On the albite (001) and (010) surfaces, the coordinatively unsaturated Al and Si centers are Lewis acidic, the coordinatively unsaturated O centers are Lewis bases, and the Lewis acids and bases on the (010) surface are stronger than those on the (001) surface.

### The mechanism of H_2_ activation by surface FLPs

We have reported in previous studies that FLPs of albite have good activity for H_2_ activation.^[28]^ In order to further analyze the microscopic mechanism of the initial, transition state and final states of H_2_ activation on the surface of albite, the current contribution contains calculated frontier orbital (Highest Occupied Molecular Orbital and Lowest Unoccupied Molecular Orbital) of H_2_ and albite (001) and (010) surfaces, namely, the energy values of (HOMO and LUMO), the density of states of the initial (IS) and transition state states (TS) activated by H_2_, and the Hirshfeld charge distribution of H_2_ dissociative adsorption. According to frontier orbital theory, in addition to HOMO and LUMO, there are Semi‐Occupied Molecular Orbital (SOMO), which may act as either HOMO or LUMO. Table 1 shows frontier orbital (HOMO, LUMO and SOMO) and energy values of H_2_ and NaAlSi_3_O_8_ surfaces. The molecular orbital of H_2_ (σ1 s)^2^ is the HOMO and (σ*1 s)^0^ is the LUMO. The empty orbital (σ3sp^3^)^0^ of LA (Al) on the surface of albite (001) is LUMO, and the orbital (σ2sp^3^)^1^ of LB(O) on the surface is SOMO. On the surface of (010), the orbitals of LA (Si) (σ3sp^3^)^1^ and LB (O) (σ2sp^3^)^1^ are both SOMO. Table 2 shows the combination mode of frontier orbital interaction between H_2_ and NaAlSi_3_O_8_ (001) and (010) surfaces. It can be seen that the SOMO on the surface of (001) and the LUMO of H_2_, that phase are different, while the HOMO of H_2_ and the LUMO on the surface, although that phase are same, but the electronegativity does not conform to the criteria, so neither combination can interact. The phase of the frontier orbital and electronegativity of HOMO of H_2_ and SOMO of surface conform to the criteria, and the energy level is the closest. When the HOMO of H_2_ with the SOMO of LB(O) interact, due to the action of FLPs electrostatic field energy, the energy level of the HOMO of H_2_ continues to rise, and the HOMO electron of hydrogen is in an excited state. At the same time, due to the characteristics of LA (Al) accepting electrons, it makes the bonding electrons of H_2_ undergo delocalization and electrons are easily excited to the empty orbital of LA (Al).

**Table 1:** Frontier orbital (HOMO,LUMO and SOMO) and energy values on H_2_ and NaAlSi_3_O_8_ surfaces.

	HOMO		LUMO		SOMO	
	Orbital	Energy value [eV]	Orbital	Energy value [eV]	Orbital	Energy value [eV]
H_2_	(σ1 s)^2^	−10.44	(σ^*^1 s)^0^	1.55	–	–
(001)	–	–	LA(σ3sp^3^)^0^	−4.90	LB(σ2sp^3^)^1^	−5.72
(010)	–	–	–	–	LA(σ3sp^3^)^1^	−5.45
					LB(σ2sp^3^)^1^	−5.47

**Table 2:** Combination modes of frontier orbital interaction between H_2_ and NaAlSi_3_O_8_(001) and (010) surfaces.

Combination	(001)			(010)		
	Frontier orbital phase	Electronegativity	ΔE [eV]	Frontier orbital phase	Electronegativity	ΔE[ eV]
H_2HOMO_/LA_LUMO_	Same	Nonconform	5.54	–	–	–
H_2HOMO_/LA_SOMO_	–	–	–	Same	Nonconform	4.99
H_2HOMO_/LB_SOMO_	Same	Conform	4.72	Same	Conform	4.97
LA_SOMO_/H_2LUMO_	–	–	–	Different	Conform	7.00
LB_SOMO_/H_2LUMO_	Different	Conform	7.27	Different	Conform	7.02

Under the synergistic action of LA (Al) and LB (O), the H−H bond is eventually broken and combined with LA (Al) and LB (O) on the surface. On the (010) surface, the HOMO of H_2_ with the SOMO of LA (Si), the SOMO of LA (Si) with the LUMO of H_2_, and the SOMO of LB (O) with the LUMO of H_2_, because both of them do not conform to the reaction conditions, they cannot interact. Only the HOMO of H_2_ with the SOMO of LB (O) meet the reaction conditions. When the two interact, similarly, under the action of the FLP's electrostatic field energy and LA(Si) electron absorption, the electrons of the HOMO hydrogen are easily excited to the half‐empty orbital of LA(Si), reducing the energy of the system. Thus it can be seen, that the activation of H_2_ on the FLPs of albite surface is a synergistic mechanism, during the H_2_ activation process, the interaction between the HOMO of H_2_ and the SOMO of LB, as well as the characteristics of LA H_2_ and the SOMO of LB, as well as the characteristics of LA accepting electron, are key to the reaction.

**Figure 8:**
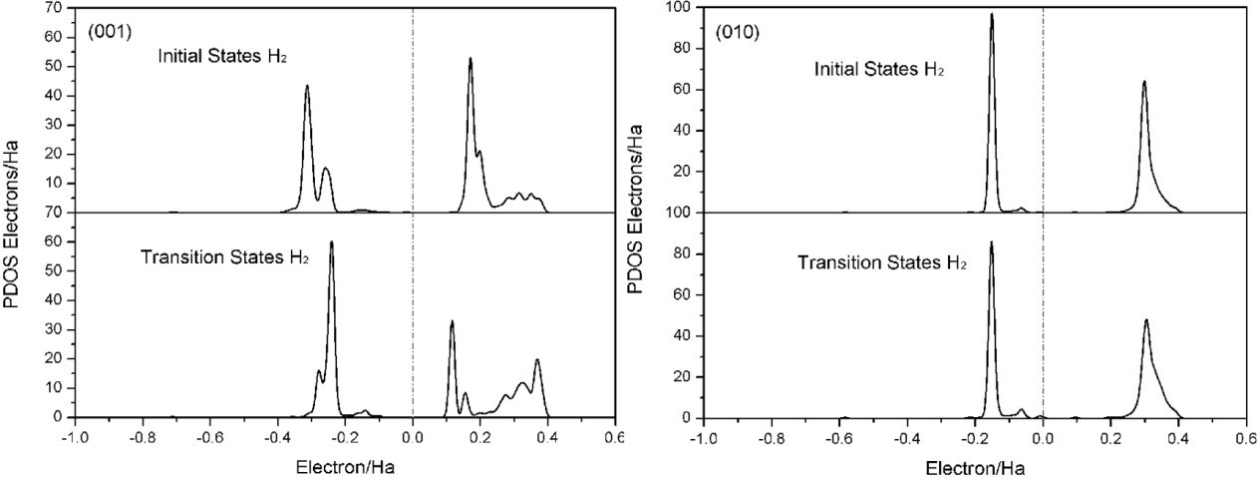
Local Density of states of initial state (IS) and transition state (TS) of H_2_ dissociation on albite (001) and (010) surfaces.

Figure 8 shows the density of states of initial state (IS) and transition state (TS) of H_2_ dissociation on the surface of albite (001) and (010). It can be seen that there are slight changes on the density of states of the TS structure of H_2_. On the (001) surface, the pseudogap slightly narrows, indicating that its structure is close to the initial state, while on the (010) surface, the pseudogap remains almost unchanged, indicating that the TS of (010) is closer to the initial state. Meanwhile, previous studies have also reported that the energy difference between TS and IS dissociated of H_2_ on the surface is not significant, and the geometric configuration of TS has little change. Therefore, the transition state of H_2_ dissociated at two surface FLPs sites can be considered as an early transition state, while the TS of (010) surface is closer to the early transition state. According to the Hammond‐Leffler hypothesis, it can be judged that the reaction of H_2_ on albite surface is an exothermic reaction, which is thermodynamically favorable. The activation energy for the dissociation of H_2_ on two surfaces is the required activation energy for H_2_ to move from outside the active region to inside the active region. Once entering the active region, dissociative adsorption will occur directly.

**Figure 9:**
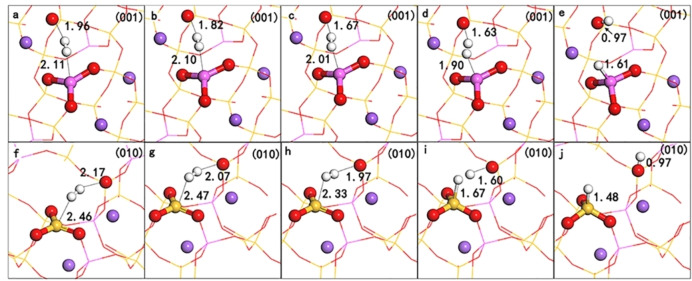
Dynamic changes in dissociative adsorption configuration of H_2_ on NaAlSi_3_O_8_ (001) and (010) surfaces.(the distance of O−H changed from 1.96 Å, 1.82 Å,1.67 Å,1.63 Å to 0.97 Å on (001), the distance of O−H changed from 2.17 Å, 2.07 Å, 1.97 Å, 1.60 Å to 0.97 Å on (010), while the distance of Al−O changed from 2.11 Å, 2.10 Å,2.01 Å, 1.90 Å to 1.61 Å, the distance of Si−O changed from 2.46 Å, 2.47 Å, 2.33 Å, 1.67 Å to 1.48 Å).

Figure 9 shows the dynamic change of H_2_ dissociative adsorption configuration on NaAlSi_3_O_8_(001) and (010) surfaces, and gives the corresponding Hirshfeld atomic charge and H−H bond parameters (see Table 3). From Figure 9 and Table 3, it can be seen that when H_2_ interacts with the surface, the H−H bond is gradually elongated, indicating that as the reaction progresses, the H−H bond is gradually weakened. From the point of view of the electron transfer state, H_2_ and LB (O) are both in the state of electron loss, while LA (Al, Si) are both in the state of electron gain, which indicates that in the reaction process, the bonding electrons of H_2_ transfer to LB (O) and LA (Al, Si) respectively, and the non bonding electrons of LB (O) transfer to the antibonding orbital of H_2_. As the reaction progresses, the H−H bond is continuously weakened until it breaks. The H^δ+^ and H^δ−^ adsorbed on LB and LA, respectively; after reaching a stable state, the charges are +0.19 e, +0.18 e and −0.16 e, −0.07 e, respectively. It indicates that H_2_ undergoes heterolysis under the synergistic effect of LA and LB.

**Table 3:** Charge distribution and H−H bond length of H_2_ dissociative adsorption.

Surface	States	Charge [e]					d_H−H_ [Å]
		LA(Si)	LA(Al)	LB(O)	H^δ+^	H^δ−^	
(001)	a	–	0.62	−0.43	0.03	−0.03	0.76
	b	–	0.62	−0.42	0.04	−0.01	0.81
	c	–	0.60	−0.40	0.05	−0.02	0.84
	d	–	0.59	−0.40	0.06	−0.02	0.85
	e	–	0.43	−0.25	0.19	−0.16	3.15
(010)	f	0.68	–	−0.43	0.03	−0.02	0.76
	g	0.68	–	−0.43	0.03	−0.02	0.79
	h	0.66	–	−0.43	0.03	0.00	0.79
	i	0.58	–	−0.39	0.10	0.00	0.87
	j	0.47	–	−0.26	0.18	−0.07	3.99

## Conclusions

As a natural resource, albite is green and environmentally friendly, and it is an ideal substrate material for the construction of solid FLPs. These results showed that coordinatively unsaturated Al and Si sites on the (001) surface and (010) surface were Lewis acidic, and the exposed O sites on the surface layers were Lewis basic. The albite ultra microchannel structure created the conditions needed for generating sterically encumbered pore openings measuring approximately 4 Å. In the reaction of H_2_ activation, the interaction between the HOMO of H_2_ and the SOMO of LB and the electron acceptance characteristics of LA are the key factors. In fact, the activation energy of H_2_ is the required activation energy from the ground state to the excited state, once the excited state is produced, dissociation adsorption of H_2_ will occur directly.

## Calculational Methods

In this paper, the DMol^3^ module^[29]^ of the MS19.1 software was used for the simulations, and DFT was used for calculations. The exchange correlation potential was described with the PBE (Perdew, Burke and Enzerhof) function under the generalized gradient approximation (GGA).[^30^, ^31^] The electron wave functions used the dual atomic orbital plus the polarization function DNP^[27]^ as the basis set, and the truncation radius was 5.2 Å. The convergence tolerances for energy, gradient and displacement were 1.0×10^−5^ Ha, 0.002 Ha/Å and 0.005 Å, respectively. In the self‐consistent functional (SCF) calculations, the convergence criterion was 1.0×10^−6^ Ha, and the smearing value was set to 0.005 Ha. Transition states were determined with the complete LST/QST^[32]^ and nudged elastic band method (NEB).^[33]^ The atomic charges were calculated with Hirshfeld population analyses.

## Conflict of interest

The authors declare no conflict of interest.

1

## Supporting information

As a service to our authors and readers, this journal provides supporting information supplied by the authors. Such materials are peer reviewed and may be re‐organized for online delivery, but are not copy‐edited or typeset. Technical support issues arising from supporting information (other than missing files) should be addressed to the authors.

Supporting Information

## Data Availability

The data that support the findings of this study are available from the corresponding author upon reasonable request.
