# Supplementary material for: Characteristics of the Frustrated Lewis Pairs (FLPs) on the Surface of Albite and the Corresponding Mechanism of H2 Activation
Source: ChemistryOpen. 2023 Oct 6;12(10):e202300058. doi: 10.1002/open.202300058 (PMC10558424; doi:10.1002/open.202300058)
Supplement: Supplementary file 1 — Supporting Information [file OPEN-12-e202300058-s001.pdf]

ChemistryOpen

Supporting Information

Characteristics of the Frustrated Lewis Pairs (FLPs) on the Surface of Albite and the Corresponding Mechanism of H_2 Activation

Yannan Zhou* and Xuegang Luo

Supplementary Table 1. The selection of the terminations of two facets

Surface	The selection of the terminations	
	Top	Thickness
(001)	0	1
(010)	0	0.5

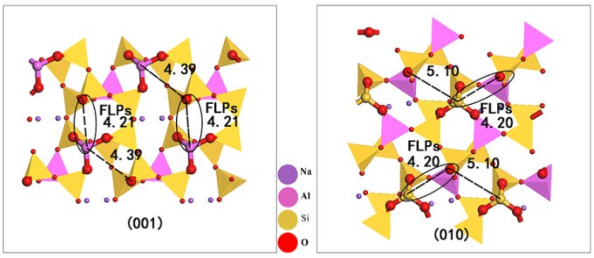

Supplementary Figure 1. Schematic diagram of albite(001) and (010) surface model

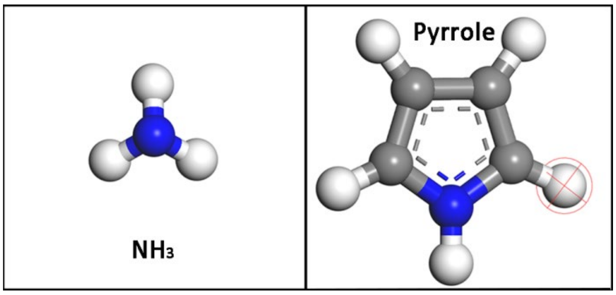

Supplementary Figure 2. Source of molecular configuration diagram of NH_3 and pyrrole

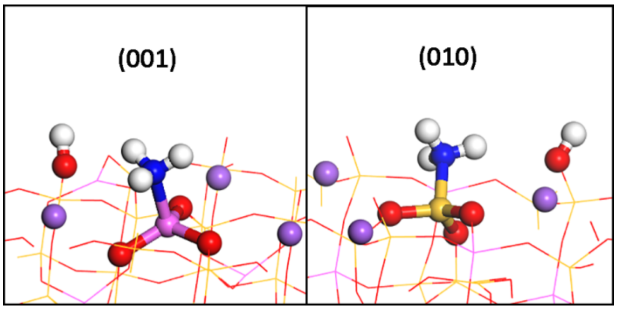

Supplementary Figure 3. Configuration diagram of LB(O) saturated with H atoms in FLPs

Supplementary Table 2. Vibration modes of infrared spectra of NH_3 on the Ab(001) and Ab(010) surfaces

Ab(001)		Ab(010)	
Frequency[cm^{-1}]	Intensity[km/mol]	Frequency[cm^{-1}]	Intensity[km/mol]
1028	168.5	1036	164.6
**1640**	**22.1**	**1628**	**20.8**
1654	21.0	1664	21.5
3344	2.2	3162	2.1
3478	2.5	3382	1.9
3512	2.9	3480	3.4

Supplementary Table 3. Vibration modes of infrared spectra of free pyrrole and pyrrole adsorbed on the two surfaces

Ab(001)		Ab(010)		Ab	
Frequency[cm^{-1}]	Intensity[km/mol]	Frequency[cm^{-1}]	Intensity[km/mol]	Frequency[cm^{-1}]	Intensity[km/mol]
585	12.5	596	6.2	454	78.4
600	6.5	616	2.9	597	0.1
621	7.7	640	12.7	616	0
669	95.3	708	106.9	647	27.5
745	42.8	787	15.7	715	142.8
807	21.4	846	2.1	785	0.6
821	66.0	858	0.5	851	1.6
861	0.8	873	2.4	858	3.1
874	0.2	931	104.4	880	0.7
1032	29.2	1031	29.5	1017	32.8
1041	28.3	1042	25	1043	32.1
1084	8.9	1081	9.2	1076	7.2
1158	2.4	1167	1.6	1135	3
1171	4.8	1186	5.5	1154	1.8
1285	3.3	1288	4.2	1270	2.3
1390	3.2	1395	4.3	1399	3
1456	1.8	1452	0	1416	5.3
1471	6.7	1480	5.1	1460	7.8
1529	8.9	1555	12.6	1521	2
3169	64.7	2873	61	3227	1.9
3209	3.6	3212	2.2	3233	4.7
3243	3.1	3217	0.9	3257	4.7
**3259**	**3.1**	**3254**	**7.1**	**3277**	**1.5**
3275	2.1	3273	2.9	3607	63.2

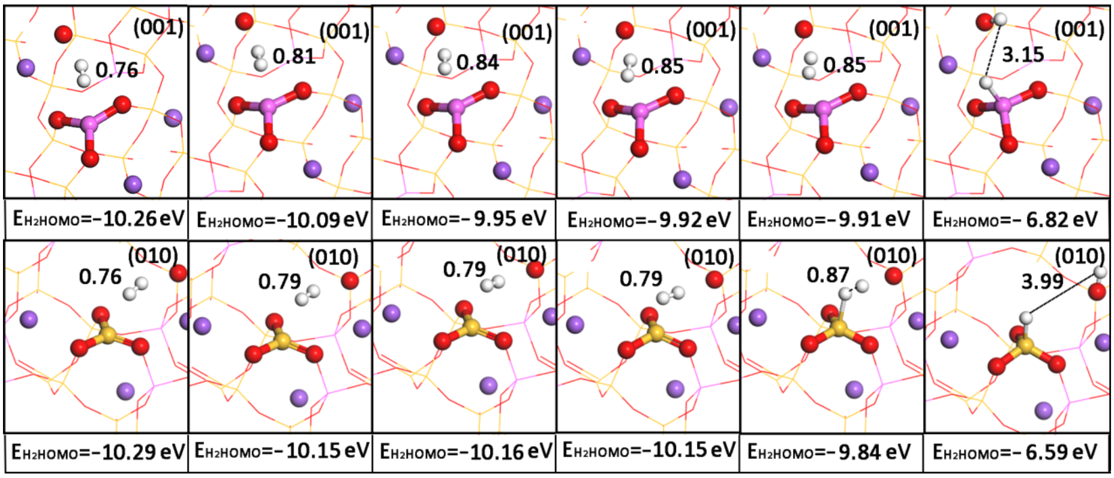

Supplementary Figure 4. Changes in HOMO energy of H_2 before dissociation at (001) and (010) surfaces

Supplementary Table 4. The imaginary frequency of transition states for H_2 dissociation

on $NaAlSi_3O_8$(001) and $NaAlSi_3O_8$(010) surfaces

Surface	Imaginary Frequency（cm^{-1}）
	TS
(001)	−185.22
(010)	−66.50
